# First Principles Investigation of Anomalous Pressure-Dependent Thermal Conductivity of Chalcopyrites

**DOI:** 10.3390/ma12213491

**Published:** 2019-10-25

**Authors:** Loay Elalfy, Denis Music, Ming Hu

**Affiliations:** 1 Materials Chemistry, RWTH Aachen University, Kopernikusstr. 10, 52074 Aachen, Germany; Music@mch.rwth-aachen.de; 2 Department of Mechanical Engineering, University of South Carolina, Columbia, SC 29208, USA

**Keywords:** thermal conductivity, pressure dependence, semiconductors, thermoelectric materials, chalcopyrites

## Abstract

The effect of compression on the thermal conductivity of CuGaS_2_, CuInS_2_, CuInTe_2_, and AgInTe_2_ chalcopyrites (space group *I*-42*d*) was studied at 300 K using phonon Boltzmann transport equation (BTE) calculations. The thermal conductivity was evaluated by solving the BTE with harmonic and third-order interatomic force constants. The thermal conductivity of CuGaS_2_ increases with pressure, which is a common behavior. Striking differences occur for the other three compounds. CuInTe_2_ and AgInTe_2_ exhibit a drop in the thermal conductivity upon increasing pressure, which is anomalous. AgInTe_2_ reaches a very low thermal conductivity of 0.2 W·m^−1^·K^−1^ at 2.6 GPa, being beneficial for many energy devices, such as thermoelectrics. CuInS_2_ is an intermediate case. Based on the phonon dispersion data, the phonon frequencies of the acoustic modes for CuInTe_2_ and AgInTe_2_ decrease with increasing pressure, thereby driving the anomaly, while there is no significant pressure effect for CuGaS_2_. This leads to the negative Grüneisen parameter for CuInTe_2_ and AgInTe_2_, a decreased phonon relaxation time, and a decreased thermal conductivity. This softening of the acoustic modes upon compression is suggested to be due to a rotational motion of the chalcopyrite building blocks rather than a compressive oscillation. The negative Grüneisen parameters and the anomalous phonon behavior yield a negative thermal expansion coefficient at lower temperatures, based on the Grüneisen vibrational theory.

## 1. Introduction

**Figure 1:**
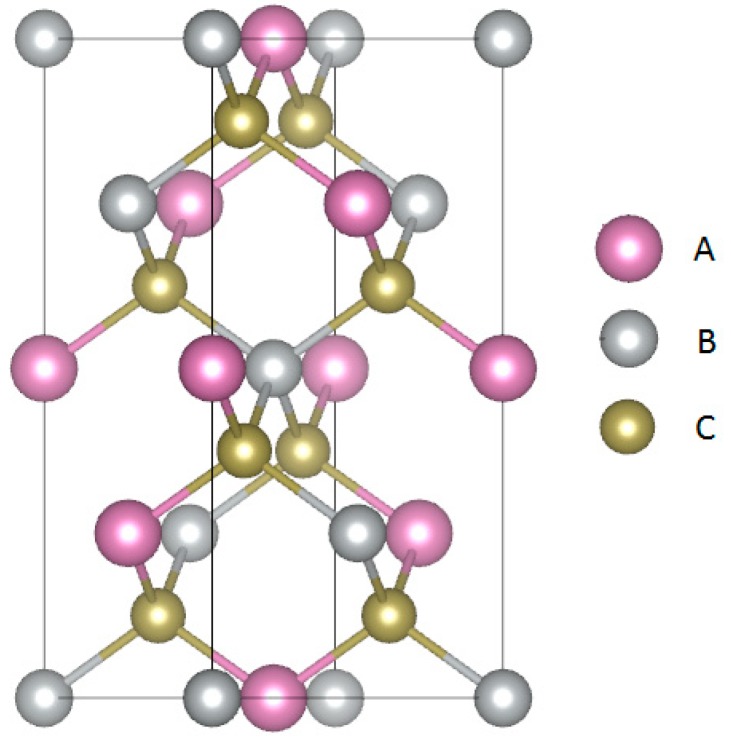
Crystal structure of the A^I^B^III^C_2_^VI^ chalcopyrite compounds.

Chalcopyrite compounds (A^I^B^III^C_2_^VI^, A^I^ = IB elements (Cu, Ag), B^III^ = IIIA elements (Al, Ga, In), C^VI^ = VIA elements (S, Se, Te), space group *I*-42*d*, as shown in Figure 1) are well-known semiconductors with a band gap in the range of 0.1 to 1 eV [1,2,3,4]. The calculated band gap for the selected systems is 1.085, 0.364, 0.469, and 0.967 eV for CuGaS_2_, CuInS_2_, AgInS_2_, and AgInTe_2_, respectively [1,2,3,4,5], being consistent with common density functional theory deviations [6]. Their structure can be derived from zincblende (ZnS) by alternating the A^I^ and B^III^ constituents at the Zn site [7]. In the zincblende structure, a Zn atom is located in a center of a tetrahedron span by S, which is equivalent to an A^I^- or B^III^-based tetrahedron span by C^VI^ [7].

These A^I^B^III^C_2_^VI^ compounds have been extensively studied for photovoltaic and thermoelectric applications [8,9,10,11,12,13]. The efficiency of thermoelectric devices depends on the thermoelectric figure of merit $ZT=TS^{2}\sigma /\kappa$, where *T* is the absolute temperature, *S* designates the Seebeck coefficient, *σ* is the electric conductivity, and *κ* stands for the thermal conductivity [14]. Other chalcopyrite compounds such as CuFeS_2_ are currently under study for their mechanical, electronic, and thermodynamic properties, being promising for thermoelectric applications [15,16]. It is well established that *κ* decreases at elevated temperatures, which is beneficial for thermoelectric applications [17]. Different studies showed that pressure has an anomalous effect on A^II^B^IV^ zincblende compounds [18,19]. Using the Slack model, Gui et al. [2] showed that *ZT* of CuInC_2_^VI^ (C^VI^ = S, Se, and Te) uniformly increases at elevated temperatures up to 850 K. While the effect of pressure on *κ* and its relation to other thermal properties has been explored, the thermal expansion coefficient [20,21,22] has not been thoroughly studied. Furthermore, atomic vibrations driving anomalous thermal behavior of CuInC_2_^VI^ compounds are not known and cannot simply be deduced from other systems. Using the quasi-harmonic Debye model, Sharma et al. have evaluated electronic, thermal, and mechanical properties of AgInC_2_^VI^ (C^VI^ = S, Se, and Te) under pressure and reported a noticeable reduction in the Grüneisen parameter and volumetric thermal expansion coefficient, as well as the bulk modulus [23].

Since the Grüneisen parameter and volumetric thermal expansion coefficient can be related to *κ* (see below for more details), it appears that pressure effects on *κ* are considerable. This is consistent with an experimental study reporting a decrease in *κ* by 30% for CuInTe_2_ under pressure up to 2.3 GPa [24]. Generally, *κ* should increase under compression [22]; however, the compounds in the current study exhibit a mixture of monotonic decrease and non-monotonic dependence under pressure. The latter phenomenon has been intensively studied recently [18,19,21]. This implies that the behavior of CuInTe_2_ and related compounds is of great interest due to non-monotonicity. The behavior of CuInTe_2_ is anomalous. Two possible mechanisms have been proposed based on experiments: (i) anharmonic behavior of lattice vibrations [24] and (ii) structural modifications under high pressure (e.g., stacking faults) [25]. The underlying physics of the *κ* reduction under compression of CuInTe_2_, and possibly other A^I^B^III^C_2_^VI^ compounds, is not fully understood.

In this work, we devise a strategy to identify the physical origin of the anomalous behavior of *κ* of A^I^B^III^C_2_^VI^ compounds under compression. Using phonon calculations, the atomic-level understanding of this anomaly is obtained by analyzing the vibrational modes and correlating these to the macroscopic observables, such as *κ*. To systematically explore the pressure effect on *κ*, CuInTe_2_ is taken as a reference, and the influence of mass, being decisive for lattice vibrations, is considered by replacing Cu with Ag, In with Ga, and Te with S within this isostructural and isoelectronic A^I^B^III^C_2_^VI^ system. Hence, CuGaS_2_, CuInS_2_, CuInTe_2_, and AgInTe_2_ chalcopyrites are explored.

## 2. Methods

In order to maximize *ZT*, *σ* and *κ* should be maximized and minimized, respectively. An increase in *σ* directly affects the total *κ* value, since *κ* = *κ_e_* + *κ_ph_*, where *κ_e_* is the electronic thermal conductivity (charge carriers also conduct heat) and *κ_ph_* is the lattice thermal conductivity. Hence, minimizing *κ_ph_* is the major route to minimize the total *κ* value. Furthermore, *κ_ph_* is likely the largest contribution for chalcopyrites since they are semiconductors. It can be obtained as follows: $\kappa_{ph}=\frac{1}{3}c_{v}v_{g}^{2}\tau$, where *v_g_* is the group velocity of phonons, *c_v_* designates the heat capacity, and *τ* is the phonon relaxation time [26]. The former two values were calculated herein from the phonon dispersion curves using the Phonopy package [27], while *τ* was obtained by solving the Boltzmann transport equation, as implemented in the ShengBTE package [28]. The isotropic approximation was applied due to isotropic pressure dependence evaluated in the current study. Harmonic and third-order interatomic force constants within three coordination shells were used as input in both packages. All interatomic force constants were generated using the Vienna Ab-initio Simulation Package (VASP) [29,30,31,32,33]. The exchange-correlation functionals were treated within the local density approximation [34], including phonon calculations. The all-electron projector augmented wave method [35] was utilized to evaluate electronic wave functions with a plane wave cutoff of 800 eV and the total energy convergence of 10^−7^ eV. No configurations were spin polarized. All structures (internal free parameters) and unit cell sizes were optimized within a force convergence condition of 10^−6^ eV·Å^−1^. A 4 × 4 × 2 *k*-mesh Monkhorst-Pack [36] was used to sample the Brillouin zone (BZ) of the 2 × 2 × 1 supercell (64 atoms) constructed from the conventional A^I^B^III^C_2_^VI^ unit cell (16 atoms, six coordination shells). Convergence tests were conducted for the *k*-mesh (from 2 × 2 × 1 to 6 × 6 × 3). At the chosen conditions (4 × 4 × 2), phonon band structure was converged and stable under pressure up to 9 GPa. Since a 10 × 10 × 10 *q*-mesh leads to a fluctuation in *κ_ph_* on the order of 10^−1^ W·m^−1^·K^−1^ (348 atomic displacements per chalcopyrite configuration accumulating Hellmann–Feynman forces for the phonon calculations), such changes are acceptable for the calculated *κ_ph_* values in the range of several W·m^−1^·K^−1^. As stated in the introduction, all A^I^B^III^C_2_^VI^ chalcopyrite compounds exhibit a band gap of >0.4 eV (see Table 1) so that only phonons are considered for the evaluation of the transport properties. The pressure dependence was modelled in the form of isotropic compressive strains in steps of 3% of the equilibrium unit cell volume (a minimum of 5 strains were considered per chalcopyrite configuration). The bulk moduli were calculated using the Rose–Vinet equation of state to evaluate the pressure associated with each strain [37]. The calculated bulk moduli for CuGaS_2_, CuInS_2_, CuInTe_2_, and AgInTe_2_ were 76.0, 64.6, 41.3, and 41.2 GPa, respectively, which is consistent with the literature [38,39,40,41,42] (see Table 1). The obtained lattice parameters were *a* = 5.387 Å and *c/a* = 1.981 for CuGaS_2_, *a* = 5.597 Å and *c/a* = 2.015 for CuInS_2_, *a* = 6.303 Å and *c/a* = 2.007 for CuInTe_2_, and *a* = 6.582 Å and *c/a* = 1.978 for AgInTe_2_. These lattice constants deviate max. 2.7% from the experimental data [43,44] (see Table 1), which is acceptable for the employed exchange-correlation functionals [6]. Finally, the quasi-harmonic approximation [45] was utilized to calculate the volumetric thermal expansion coefficient due to its relationship with *κ_ph_*, as detailed below.

## 3. Results and Discussion

### 3.1. Lattice Thermal Conductivity

**Figure 2:**
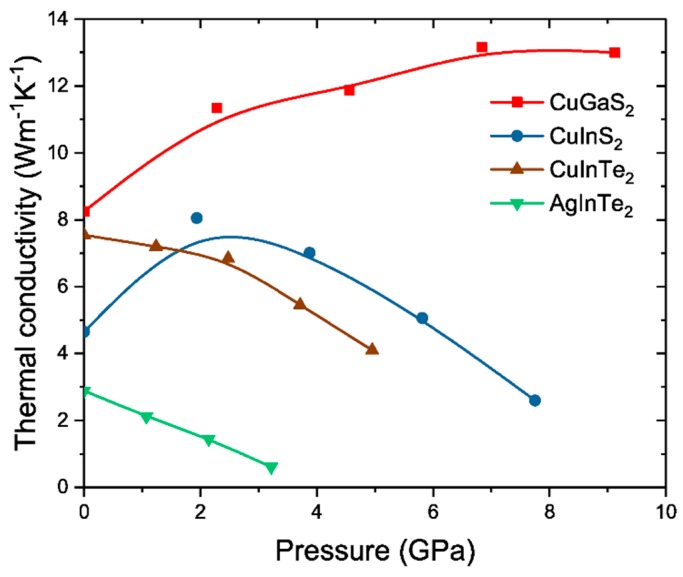
Thermal conductivity at 300 K for CuGaS_2_, CuInS_2_, CuInTe_2_, and AgInTe_2_ under compression. The solid lines connecting the data points serve as a guide to the eye.

In Figure 2, the lattice thermal conductivity of all A^I^B^III^C_2_^VI^ compounds studied in this work is plotted against an increasing pressure. First, the ambient pressure conditions (0 GPa) are discussed. CuGaS_2_ exhibits a relatively large *κ_ph_* value of 8.2 W·m^−1^·K^−1^, which deviates 12% from the measured one (9.3 W·m^−1^·K^−1^) [46]. In the case of CuInS_2_, *κ_ph_* reaches 4.6 W·m^−1^·K^−1^. The *κ_ph_* value of AgInTe_2_ is 2.9 W·m^−1^·K^−1^, which is only 7% offset from the experimental value of 2.7 W·m^−1^·K^−1^ [47]. CuInTe_2_ attains 7.6 W·m^−1^·K^−1^, which is an 18% deviation compared to the experimentally obtained value of 6.2 W·m^−1^·K^−1^ [48]. Hence, these differences are acceptable based on the *κ_ph_* deviations of other theoretical studies from measurements [46,49,50]. See Table 1 for comparison. Furthermore, based on a comparison of predicted and experimental *κ_ph_* values for diamond, SiC, GaN, Si, GaAs, InSb, SrTiO_3_, and PbTe, theoretical data at elevated temperatures commonly overestimate the measured values [51], so that the calculated low *κ_ph_* data in this study should be even lower under ordinary experimental conditions (e.g., presence of defects). It is clear that trends are properly captured within the methodology used in the current study.

Striking differences were obtained between the *κ_ph_* behavior of CuGaS_2_, CuInS_2_, CuInTe_2_, and AgInTe_2_ under compression (see Figure 2). The pressure range explored herein can be reached in samples synthesized by non-equilibrium vapor phase condensation processes [52]. The *κ_ph_* value of CuGaS_2_ always increases with pressure up to 9.5 GPa. This is a common behavior for most compounds [22]. A drastically different dependence is obtained as soon as the heavier In is considered instead of Ga (B^III^ in A^I^B^III^C_2_^VI^). Under pressure up to 6 GPa, the *κ_ph_* of CuInTe_2_ decreases from 7.6 to 4.1 W·m^−1^·K^−1^, which is anomalous. This is consistent with the experimental data [24,25], implying that important physics is captured within the methodology employed herein and structural modulations are not indispensable to drive the anomaly. By exchanging Te with lighter S (C^VI^ in A^I^B^III^C_2_^VI^) and hence forming CuInS_2_, *κ_ph_* increases up to 2 GPa, which is again a common behavior and equivalent to that of CuGaS_2_. Upon a further pressure increase, *κ_ph_* begins to decrease and reaches a slightly lower value at 8 GPa than that at 0 GPa. To account for the effect of the transition metal constituent (A^I^ in A^I^B^III^C_2_^VI^), Cu in CuInTe_2_ is exchanged with the heavier Ag. AgInTe_2_ exhibits a significantly lower *κ_ph_* value and a steeper decrease in *κ_ph_* under pressure, reaching 0.2 W·m^−1^·K^−1^ at 2.6 GPa. This is a very low value for *κ_ph_* and is comparable to that of some polymers, such as polytetrafluoroethylene [53]. Such low *κ_ph_* values under pressure should lead to an enhanced thermoelectric performance, according to several studies [54,55,56].

### 3.2. Acoustic Phonon Dispersion

**Figure 3:**
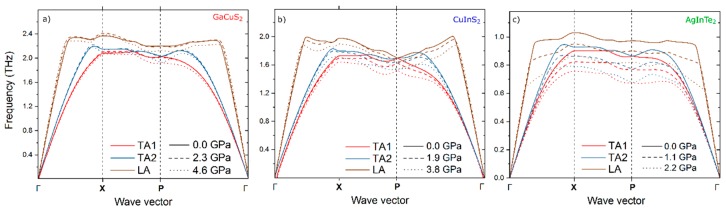
Acoustic phonon dispersion at different pressures of (**a**) CuGaS_2_, (**b**) CuInS_2_, and (**c**) AgInTe_2_. TA1 and TA2 are two transverse acoustic modes, and LA is a longitudinal acoustic mode.

Since *κ_ph_* and all the corresponding factors, *v_g_*, *c_v_*, and *τ*, are governed by phonons, phonon dispersion curves are further discussed to explain the anomalous behavior. The acoustic phonon modes at 300 K contribute 85%, 70%, 60%, and 80% of *κ_ph_* for CuGaS_2_, CuInS_2_, CuInTe_2_, and AgInTe_2_, respectively, so that non-locality of the exchange-correlation functional is of less significance since it would mainly contribute to optical phonon frequencies [57]. Therefore, in this study, the behavior of acoustic phonon modes and the effect of pressure thereon are considered in detail. Since AgInTe_2_ and CuInTe_2_ exhibit the same *κ_ph_* behavior, whereby the former undergoes a more drastic change, AgInTe_2_ is taken as a representative. Figure 3 contains the acoustic phonon modes under different pressures for CuGaS_2_ (common *κ_ph_* behavior), CuInS_2_ (intermediate case), and AgInTe_2_ (anomalous case). The effect of pressure on the phonon dispersion curves for different compounds is noticeably different. For CuGaS_2_ (Figure 3a), all acoustic transverse (TA) and longitudinal (LA) modes are not significantly affected by pressure. In the case of AgInTe_2_ (Figure 3c), the phonon modes are considerably softened over the entire BZ. The phonon modes of CuInS_2_ (Figure 3b) do not behave uniformly. The TA modes of CuInS_2_ are similar to the behavior of AgInTe_2_ after crossing a pressure threshold, and the LA modes are more like those of CuGaS_2_.

The atomic vibrations were analyzed from the phonon dispersion curves using a tool developed by Miranda et al. [58]. For the phonon modes with decreasing frequency upon compression, e.g., LA of AgInTe_2_ starting at (0, 0, 0.3) on the Γ-X and X-P path (30% of the path length), as shown in Figure 3c, the vibrations are unconventional. The metal-centered tetrahedra (A^I^ in A^I^B^III^C_2_^VI^) oscillate in the manner that the angles between neighboring units are changing, while the bond lengths are fixed. However, the conventional thermal vibrations occur in the form of a compression wave, where the bonds are mostly stretched, as in the case of CuGaS_2_. The anomalous behavior is due to the circular motion of the metal atoms (A^I^ = Cu or Ag) around the equilibrium position. The anomalous chalcopyrites tend to keep the bond length fixed and the excitations appear in the form of bond bending. Such behavior is consistent with the tension effect introduced by Dove et al. [59], showing that in the case where the energy required for stretching is too high, a finite transverse displacement in the centers of polyhedra occurs, resulting in rotation.

### 3.3. Phonon Relaxation Time

**Figure 4:**
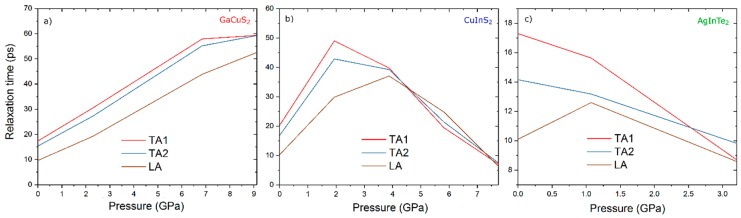
Phonon relaxation time under pressure of (**a**) CuGaS_2_, (**b**) CuInS_2_, and (**c**) AgInTe_2_. TA1 and TA2 are two transverse acoustic modes, and LA is a longitudinal acoustic mode.

A decrease in the phonon frequency upon compression (softening of the acoustic phonon modes) implies the negative Grüneisen parameter (*γ*), which can be related to *τ* (1/*τ* = *γ*^2^ within the Debye–Callaway model [60]) one of the three physical variables describing *κ_ph_*. Since *v_g_*^2^ decreases uniformly with pressure for all A^I^B^III^C_2_^VI^ compounds explored in this work and *c_v_* is affected by only 0.1%, it appears that *τ* is the major constituent responsible for the anomaly. Hence, *τ* is further explored for CuGaS_2_, CuInS_2_, and AgInTe_2_ as a function of pressure. The corresponding *τ* data for the acoustic phonon modes are shown in Figure 4. For CuGaS_2_ (Figure 4a), *τ* increases up to 300% with pressure. This implies that the absolute value of *γ* decreases. For CuInS_2_ (Figure 4b), *τ* increases up to 2 GPa in the case of TA and further to 4 GPa in the case of LA and TAs and then decreases, which is supported by the fact that *γ* changes the sign for a number of low-frequency phonons as appearing in the dispersion relation in Figure 3b. For AgInTe_2_ (Figure 4c), *τ* decreases in the whole pressure range and hence *γ^2^* is increasing, while the values are negative (softening of the acoustic phonon modes). Therefore, an increasing *τ* with pressure gives rise to an increasing *κ_ph_*, as in the case of CuGaS_2_. Anomalous *κ_ph_* of AgInTe_2_ exhibits a small *τ* value and negative *γ*. The notion of negative *γ* values and softening of the acoustic phonon modes is consistent with the literature on AgGaS_2_ [61]. In the present work, an important step is made towards a relationship with *κ_ph_*.

### 3.4. Thermal Expansion Coeficient

**Figure 5:**
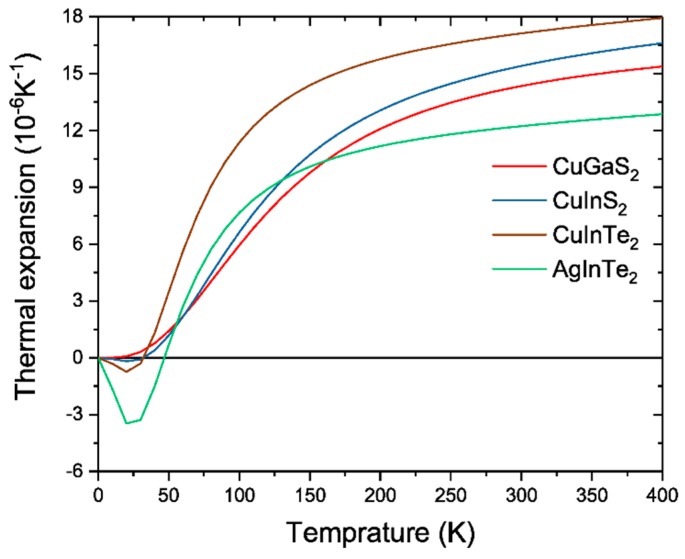
Volumetric thermal expansion coefficient at ambient pressure for CuGaS_2_, CuInS_2_, CuInTe_2_, and AgInTe_2_.

According to the Grüneisen vibrational theory of thermal expansion [59], negative *γ* yields a negative volumetric thermal expansion coefficient (α) since $\gamma =\alpha V/Bc_{v}$, where V is the volume and B stands for the bulk modulus. As *α* can be obtained independently from *γ* (quasi-harmonic approximation was used herein), probing *α* is important. Furthermore, this may help other experimentalists, besides those focusing on thermoelectric devices, to critically appraise the results obtained in this study. Therefore, in Figure 5 the *α* value is plotted at different temperatures. For CuGaS_2_, having an increasing *κ_ph_* value under pressure, *α* is always positive. CuInS_2_ possesses a slightly negative *α* value. However, both CuInTe_2_ and AgInTe_2_ exhibit negative *α* at low temperatures. The pressure dependence on *α* is not shown here since the major effect is an offset of the negative *α* region to higher temperatures, conserving the trends between these chalcopyrites. The anomalous behavior of CuInTe_2_ and AgInTe_2_ is thus driven by softening of the acoustic phonon modes under compression, leading to negative *α* and *γ*.

## 4. Conclusions

The thermal conductivity of CuGaS_2_, CuInS_2_, CuInTe_2_, and AgInTe_2_ A^I^B^III^C_2_^VI^ chalcopyrites exhibits a different behavior under pressure, ranging from increasing (CuGaS_2_), as for most compounds, alternating (increasing and decreasing for CuInS_2_), and to decreasing, as in the case for CuInTe_2_ and AgInTe_2_, which is anomalous. This can be understood based on the phonon dispersion curves. Softening of the acoustic phonon modes occurs for these anomalous chalcopyrites. This leads to the negative Grüneisen parameter and negative volumetric thermal expansion coefficient. The decrease in phonon frequency upon compression is suggested to be due to the phonon oscillations in the form of a rotational motion rather than compressive waves. The physical origin of the anomalous thermal conductivity is thus identified in this work in terms of higher-order phonon—phonon interactions, and AgInTe_2_ with a very low thermal conductivity of 0.2 W·m^−1^·K^−1^ at 2.6 GPa is proposed to be a promising thermoelectric compound.

## Figures and Tables

**Table 1:** Comparison between calculated and reported values for band gap, bulk modulus, lattice parameters, and thermal conductivity.

	Band Gap ^1^ (eV)		Bulk Modulus ^2^ (GPa)		Lattice Parameters ^2^ *a* (Å), *c/a*		*κ*^2^ (W/mK)	
	This Work	Ref.	This Work	Ref.	This Work	Ref. [41]	This Work	Ref.
CuGaS_2_	1.085	0.92 [1]	76.0	94 [39]	5.387, 1.981	5.34, 1.95	8.2	9.3 [46]
CuInS_2_	0.364	0.35 [1]	64.6	75 [40]	5.597, 2.015	5.51, 2.00	4.6	-
CuInTe_2_	0.469	0.02–0.91 [2]	41.3	45 [41]	6.303, 2.007	6.16, 2.00	2.9	2.7 [47]
AgInTe_2_	0.976	0.91 [5]	41.2	41.1 [42]	6.582, 1.978	6.4, 1.96	7.6	6.2 [48]

^1^ Band gaps are compared with computational results at 0 K, except for AgInTe_2_. ^2^ Comparison with experimental values at room temperature.
